# Pre-Exposure With Extracellular Vesicles From *Aspergillus fumigatus* Attenuates Inflammatory Response and Enhances Fungal Clearance in a Murine Model Pulmonary Aspergillosis

**DOI:** 10.3389/fcimb.2022.898619

**Published:** 2022-06-01

**Authors:** Jéssica Amanda Marques Souza, Isabella Luísa da Silva Gurgel, Nathália Luísa Sousa de Oliveira Malacco, Flávia Rayssa Braga Martins, Celso Martins Queiroz-Junior, Mauro Martins Teixeira, Frederico Marianetti Soriani

**Affiliations:** ^1^ Centro de Pesquisa e Desenvolvimento de Fármacos, Departamento de Genética, Ecologia e Evolução, Instituto de Ciências Biológicas, Universidade Federal de Minas Gerais, Belo Horizonte, Brazil; ^2^ The Lopes Lab, Institute of Parasitology, McGill University, Montreal, QC, Canada; ^3^ Centro de Pesquisa e Desenvolvimento de Fármacos, Departamento de Morfologia, Instituto de Ciências Biológicas, Universidade Federal de Minas Gerais, Belo Horizonte, Brazil; ^4^ Centro de Pesquisa e Desenvolvimento de Fármacos, Departamento de Bioquímica e Imunologia, Instituto de Ciências Biológicas, Universidade Federal de Minas Gerais, Belo Horizonte, Brazil

**Keywords:** extracellular vesicles, filamentous fungus, *Aspergillus fumigatus*, immunization, host-pathogen interactions

## Abstract

*Aspergillus fumigatus* is a ubiquitous and saprophytic filamentous fungus and the main etiologic agent of aspergillosis. Infections caused by *A. fumigatus* culminate in a strong inflammatory response that can evolve into respiratory failure and may be lethal in immunocompromised individuals. In the last decades, it has been demonstrated that extracellular vesicles (EVs) elicit a notable biological response in immune cells. EVs carry a variety of biomolecules, therefore are considered potential antigen delivery vehicles. The role of EVs as a strategy for modulating an effective response against infections caused by *A. fumigatus* remains unexplored. Here we investigate the use of EVs derived from *A. fumigatus* as an immunization tool to induce a more robust immune response to *A. fumigatus* pulmonary infection. In order to investigate that, male C57BL/6 mice were immunized with two doses of EVs and infected with *A. fumigatus*. Pre-exposure of mice to EVs was able to induce the production of specific IgG serum for fungal antigens. Besides that, the immunization with EVs reduced the neutrophilic infiltrate into the alveoli, as well as the extravasation of total proteins and the production of proinflammatory mediators IL-1β, IL-6, and CXCL-1. In addition, immunization prevented extensive lung tissue damage and also improved phagocytosis and fungus clearance. Noteworthy, immunization with EVs, associated with subclinical doses of Amphotericin B (AmB) treatment, rescued 50% of mice infected with *A. fumigatus* from lethal fungal pneumonia. Therefore, the present study shows a new role for *A. fumigatus* EVs as host inflammatory response modulators, suggesting their use as immunizing agents.

## Introduction

The Aspergillus genus comprises ubiquitous and saprophytic fungi that play an important role in the environmental recycling of carbon and nitrogen (Latgé, 1999). It includes about 200 species, some of which are considered important pathogens in humans. Among them, *Aspergillus fumigatus* is the main causative agent of infections, mainly in immunocompromised individuals (Dagenais and Keller, 2009; Kim, 2016). The main clinical complications caused by this fungus are allergic bronchopulmonary aspergillosis, aspergilloma, and invasive pulmonary aspergillosis (IPA), the latter being one of the most serious complications (Dagenais and Keller, 2009). The mortality rates associated with IPA vary from 50% to 90% (Kousha et al., 2011; Szalewski et al., 2018). *A. fumigatus* infection occurs by conidia inhalation, followed by its deposition in bronchioles and alveolar space. The production of inflammatory mediators by alveolar macrophages leads to the effector cells’ recruitment of neutrophils. Neutrophils avoid the hyphae invasion, but on the other hand they create a highly inflammatory environment, which if not controlled can result in tissue damage, loss of function, and can also be fatal **(** Dagenais and Keller, 2009 **;** Malacco et al., 2019b **).**

Over the past few years, there has been a considerable expansion in the research of antifungal therapies directed against aspergillosis (Walsh et al., 2008; Patterson and Strek, 2014). Although antifungal drugs are effective, they can trigger serious side effects such as nephrotoxicity and hepatotoxicity, which are considered limiting factors for treatment. In addition, the emergence of drug-resistant infections represents another challenge (Odds et al., 2003; Howard et al., 2009). Despite the fact that fungi cause systemic mycoses which have a deep impact on human health, fungal diseases are neglected (Rodrigues and Nosanchuk, 2020). Thus, there is a critical demand for prophylactic options and therapeutic alternatives that are safer and more effective in preventing and treating aspergillosis.

Recently, extracellular vesicles (EVs) have gained a lot of attention as they provide an innovative pathway for safe and effective delivery of antigenic material (Mehanny et al., 2021). It is well established that EVs carry a variety of biomolecules such as proteins, lipids, carbohydrates, and nucleic acids, and therefore are considered an important mechanism of intercellular communication (Yoon et al., 2014). Previous studies have revealed that pathogenic prokaryotic and eukaryotic organisms release virulence factors from EVs, and therefore are considered potent stimulators of the immune system (Brown et al., 2015; Joffe et al., 2016). The first evidence that fungal EVs are able to stimulate the immune system was identified in *Cryptococcus neoformans* (Rodrigues et al., 2007; Rodrigues et al., 2008; Oliveira et al., 2010). Since then, *in vitro* studies have shown that fungal EVs can stimulate the inflammatory mediator’s production, impact fungal clearance mechanisms, and influence macrophage polarization (Vargas et al., 2015; Silva et al., 2016; Bitencourt et al., 2018; Ikeda et al., 2018; Souza et al., 2019; Brauer et al., 2020).

The pretreatment with EVs in an invertebrate model of *Galleria mellonella* larvae stimulated a protective response against a lethal challenge with *Candida albicans*, *C. neoformans*, and the filamentous fungus *Aspergillus flavus* (Vargas et al., 2015; Colombo et al., 2019; Vargas et al., 2020; Brauer et al., 2020). Interestingly, studies using murine models demonstrated that pretreatment with EVs from *C. neoformans* was able to stimulate a strong antibody response and prolong the survival of infected animals (Rizzo et al., 2021). Additionally, recent studies have shown that immunization with EVs from *C. albicans* and *Paracoccidioides brasiliensis*, associated with adjuvants, is able to modulate the production of inflammatory mediators, influence fungal clearance, promote antibody production, and consequently confer protection (Vargas et al., 2020; Baltazar et al., 2021).

Recently, our research group has shown that *A. fumigatus* is able to release EVs that carry a variety of biomolecules involved in the physiology and pathogenicity of the microorganism. Besides that, we have demonstrated that *A. fumigatus* EVs are able to stimulate macrophages and neutrophils *in vitro*, promoting the production of TNF-α and CCL2 and stimulating fungal clearance (Souza et al., 2019). Given those properties, we hypothesized that *A. fumigatus* EVs could induce the activation of the host’s immune system and generate a protective response against *A. fumigatus* infection. Here, we investigated the immunization effects of *A. fumigatus* EVs after infection by the fungus. Interestingly, our results demonstrate that multiple immunizations with *A. fumigatus* EVs result in a mild inflammation after infection, with lower levels of important mediators and a decrease of neutrophils infiltration into lungs. This more controlled inflammatory response results in higher fungal clearance and lung tissue protection. Moreover, we demonstrate the potential synergistic effect of EVs immunization in aspergillosis treatment.

## Materials And Methods

### Mice

Male C57BL/6 mice (8-10 weeks old) were obtained from the central animal facility of Universidade Federal de Minas Gerais. Mice were maintained with free access to commercial chow and water. All procedures described were approved by the local animal ethics committee (Comitê de Ética no Uso de Animais - CEUA 255/2018).

### EVs Isolation

The isolation of EVs was performed according to Souza and colleagues (Souza et al., 2019). 2 × 10^8^ conidia from *A. fumigatus* A1163 strain were inoculated in 1 L of YG medium (0.5% w/v yeast extract powder; 2% w/v glucose; 0.1% v/v trace elements). Conidia were incubated for 48 hours under 120 rpm at 37°C. The mycelium was separated from supernatant by filtration using paper filter. Supernatant was filtered using a 0.45 µm filter (Sartorius) and concentrated up to 25 mL of Amicon ultra-concentration system (cutoff 100 KDa, Millipore). The concentrated supernatant was centrifuged at 100,000 *g* for 1 hour at 4°C. The pellet of EVs was washed with phosphate-buffered saline 1X (PBS) and centrifuged in the same conditions. EVs pellet was resuspended in 260 µL of saline, treated with Protease Inhibitor Cocktails 10X (Sigma) in 1:100 and stored at −80°C.

### Fungal Culture Conditions

Reactivated conidia of *A. fumigatus* A1163 strain were seeded in Petri dishes containing complete medium (YAG) (0.5% w/v yeast extract powder; 2% w/v glucose; 2% w/v agar and 100 μL of trace elements) and incubated in an oven at 37°C for 48 hours. After the incubation period, a conidia suspension was prepared in PBS 1X. The suspension was centrifuged at 1,400* g* for 10 minutes. The conidia pellet was resuspended in PBS 1X and the concentration was determined after counting in a Neubauer chamber.

### Experimental Strategy

Mice were anesthetized with 3% isoflurane/oxygen steaming mixture and then were intranasally immunized with 2.2 × 10^9^ EVs (equivalent to 0.4 µg of total protein present in EVs, as described by Souza et al., 2019) in 40 µL of saline. Two immunizations were given at an interval of 7 days each, as previously described (Ikeda et al., 2018). One week after the last immunization the animals were intranasally infected with 1 x 10^8^ *A. fumigatus* conidia. Forty-eight hours post infection, the animals were euthanized by anesthetic overdose with ketamine/xylazine (240 mg/kg and 30 mg/kg, respectively, i.p.) and blood, bronchoalveolar lavage (BAL), and lungs were collected to evaluate the antibodies production and inflammatory response. The groups were classified into non-immunized and non-infected (Mock), non-infected that received EVs (EVs), infected (Afu – *A. fumigatus*) and mice that received EVs and were infected (EVs+Afu).

To evaluate survival rates, the animals were immunized and infected as described above. Animals were treated intraperitoneally with 250 µg/kg of Amphotericin B (AmB) 6, 24, 48, 72, and 96 hours post infection. The animals were monitored for 7 days. The groups were classified as infected (Afu); mice that received EVs and were infected (EVs+Afu); infected that received Amphotericin B (Afu+AmB); and mice that received EVs, were infected and received Amphotericin B (EVs+Afu+AmB).

### Bronchoalveolar Lavage (BAL) and Tissue Extraction

Mice were euthanized with a lethal dose of ketamine/xylazine (240 and 30 mg/kg, respectively, i.p.), blood was collected from the abdominal vena cava for antibodies levels (serum) evaluation, and BAL was performed. For that, the mice trachea was exposed, a 1.7-mm catheter was inserted, and two aliquots of 1 mL of PBS 1X were flushed three times into the bronchoalveolar compartment to recover the leukocytes and fungi in the airways of mice. The cells were centrifuged and resuspended in 100 µL of PBS 1X and used to total (Neubauer chamber) and differential cell counts and fungi counts. Cytocentrifuge preparations (Shandon III) stained with Panoptic stain (Laborclin) were used for differential counts of leukocytes, based on morphological criteria.

Pulmonary tissue was extracted after perfusion of 5 mL of cold sterile PBS 1X in order to remove circulating blood. The right lung of mice was collected for indirect quantification of neutrophils (myeloperoxidase assay - MPO), macrophages (N-acetyl-beta-D-glucosaminidase - NAG), and eosinophils (eosinophil peroxidase - EPO) recruited into the tissue. The left lobe of the lungs was fixed in buffered formalin 10% for 48 hours, for further histological examination.

### Phagocytosis and Fungal Killing

The phagocytosis of the conidia was evaluated in the cytospin slides containing Panoptic stained cells from BAL. The phagocytic index was determined as the percentage of cells presenting at least 1 conidium phagocyted over total counted cells. This ratio was calculated as a mean of 300 cells counted in each slide. To assess fungal killing, 10 µL of centrifuged BAL was submitted to serial dilution and was plated in YAG. Plates were incubated at 37°C and colonies were counted after 16 hours for determining the colony-forming units (CFU).

### Measurement of Inflammatory Mediators and Total Protein

BAL fluid supernatants were used for cytokine/chemokines (TNF-α, IL-1β, IL-6, IL-17, C5a and CXCL1) evaluation by ELISA, according to the manufacturer’s instructions (R&D Systems, USA), and total protein quantification, using Bradford assay (BioRad).

### Measurement of EVs-Reactive Antibody Titer

Specific IgG for EVs was evaluated in the serum of immunized (EVs) and non-immunized (Mock) mice. 100 ng of EVs were used to sensitize the wells in a 96-well plate overnight. Then, the wells were washed and blocked with PBS 1X/1% BSA (w/v). After washing, the plate was incubated with mouse serum diluted 1:10 in PBS 1X/0.1% (v/v) BSA. The plate was washed and incubated with anti-mouse IgG (Southern Biotech) and then incubated with streptavidin-HRP (R&D Systems). After the last wash, the reaction was carried out by adding o-Phenylenediamine dihydrochloride (OPD–Sigma) in citrate buffer (pH = 5) containing H_2_O_2_. Plates were read at a wavelength of 492 nm.

### Myeloperoxidase (MPO), N-acetyl-beta-D-glucosaminidase (NAG), and Eosinophil Peroxidase (EPO) Quantification

For indirect quantification of neutrophils, macrophages, and eosinophils in lung tissue, the levels of MPO, NAG, and EPO were evaluated, respectively. Assays were performed as previously described (Russo et al., 2009; Malacco et al., 2019a; Malacco et al., 2019b). To assess MPO activity, 50 milligrams of lung tissue were homogenized in a buffered solution containing antiproteases. **The sample was centrifuged and the pellet formed was homogenized with a 0.2% NaCl solution** (w/v) **and a 1.6% NaCl solution supplemented with 5% glucose** (w/v)**. The sample was centrifuged and the pellet resuspended in a buffer of 0.05 M Na_3_PO_4_ and 0.5% hexadecyltrimethylammonium bromide** (w/v) **(HTAB; Sigma).** The suspension was submitted to three freeze-thaw steps using liquid nitrogen. **Assays were performed in 96-well plates.** MPO levels were accessed using 25 μL of the supernatant of the homogenized sample and 25 µL of a solution of 1.6 mM of 3,39-5,59-tetramethylbenzidine (TMB; Sigma—dissolved in dimethyl sulfoxide - DMSO) more 0.002% of H_2_O_2_, diluted in phosphate buffer (pH 5.4) containing HTAB. The reaction was stopped by the addition of 1 M H**_2_** SO**_4_** (v/v). The absorbance was measured at a wavelength of 450 nm.

**To access NAG activity, the tissue was initially homogenized with the same previously mentioned buffer.** The sample was centrifuged and the pellet formed was homogenized with a 0.2% NaCl solution (w/v) and a 1.6% NaCl solution supplemented with 5% glucose (w/v). The pellet obtained was resuspended in a solution of 0.9% NaCl (w/v) containing 0.1% of Triton X-100 (v/v). NAG levels were accessed using 100 µL of homogenized supernatant and 100 µL of a solution of citrate/phosphate buffer 0.1 M (w/v) (pH 4.5) containing 1 mM of p-nitrophenyl-N-acetyl-beta-D-glucosaminide (Sigma). The reaction was stopped by the addition of 0.2 M glycine buffer (w/v) (pH 10.6). The absorbance was measured at a wavelength of 405 nm.

To access EPO activity the tissue was homogenized with PBS 5X (w/v). The sample was centrifuged and the pellet formed was homogenized with a 0.2% NaCl solution (w/v) and a 1.6% NaCl solution supplemented with 5% glucose (w/v). The pellet obtained was resuspended in a solution of PBS 1X with HTAB 0.5% (w/v). EPO levels were accessed using 75 µL of homogenate supernatant and 75 µL of o-Phenylenediamine (OPD; Sigma) solution dissolved in Tris-HCl Buffer and 30% H_2_O_2_. The reaction was stopped by the addition of 1 M H_2_SO_4_ (v/v). The absorbance was measured at a wavelength of 492 nm.

### Histological Analysis

To assess lung damage followed by *A. fumigatus* infection, the left lobes of the lungs, collected after bronchoalveolar lavage, were formalin-fixed buffered 10% for 48 hours, after that they were gradually dehydrated in ethanol and embedded in paraffin. About 5 µm sections were cut and stained with H&E for examination under light microscopy. The histopathological score was performed by a pathologist blinded to the experimental procedure and severity and distribution of inflammatory damage. The total pathology score is the sum of the severity and the distribution and potentially range from 0 to 10. The severity scores are: none = 0; minimal = 1; mild = 2; moderate = 3; marked = 4; severe = 5. The distribution scores are: none = 0; focal = 1; locally extensive = 2; multifocal = 3; multifocal and coalescent = 4; diffuse = 5 (Malacco et al., 2019a).

### Statistical Analysis

Statistics were performed using GraphPad Prism 8.0. One-way ANOVA, followed by Tukey’s post-test was used to compare more than two groups, and unpaired t-test was used for comparisons between two groups. The survival curves were analyzed by Log-rank test. Results with P < 0.05 were considered statistically significant and they are shown as means ± standard deviation (SD).

## Results

### Immunization With *A. fumigatus* EVs Decreases Inflammatory Infiltrate for Lungs Post Infection

**Figure 1:**
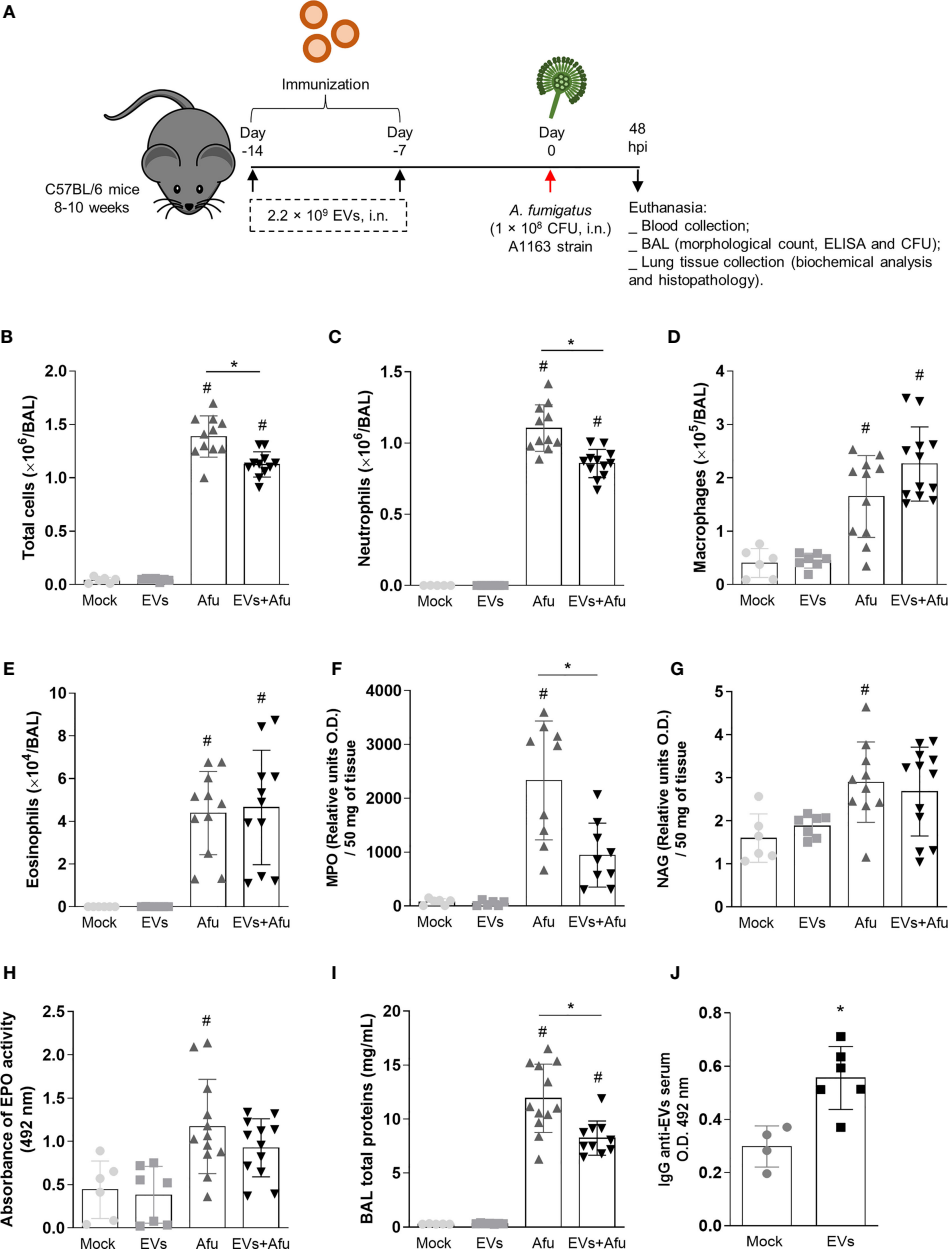
Immunization with *A. fumigatus* EVs stimulates the production of antibodies and modulate inflammatory infiltrate to lungs during infection. C57BL/6 WT mice were immunized twice with EVs (2.2 × 10^9^ EVs/immunization, i.n.), challenged intranasally with 1 × 10^8^ *A. fumigatus* conidia 14^th^ day and euthanized 48 h later (schematic protocol in **(A)**. BAL was harvested to quantify numbers of leukocytes **(B)**, neutrophils **(C)**, macrophages **(D)** and eosinophils **(E)**. MPO **(F)**, NAG **(G)** and EPO **(H)** activity in lung tissue. The total protein level in BAL is shown in **(I)**. Blood was collected to assess the production of IgG reactive to EVs **(J)**. Data are mean ± SD of N = 5-6 animals per group. #/* P < 0.05 when compared to the Mock group (#) or as indicated (*), as determined by One Way-ANOVA and two-sample independent t-tests. Data are representative of two experiments.

Infections caused by *A. fumigatus* culminates in a strong inflammatory response that can evolve into respiratory failure and may be lethal (Malacco et al., 2019a; Malacco et al., 2019b; Malacco et al., 2020). In order to evaluate if *A. fumigatus* EVs have the potential to stimulate a modulatory and effective immune response to control infection, mice were intranasally immunized with EVs and infected with *A. fumigatus* conidia. Forty-eight hours post infection the clinical parameters, as well as inflammatory and immunological parameters, were evaluated (**Figure 1A**). It was observed that, during infection, mice immunized with *A. fumigatus* EVs showed a decrease of 20% in total cell infiltrate into the airways compared to non-immunized animals (**Figure 1B**). This decrease of inflammatory cell infiltrate into the alveoli was characterized, mainly, by a reduction in neutrophil influx (**Figure 1C**). Macrophages and eosinophils infiltrates were in similar levels (**Figures 1D, E**).

We also evaluated inflammatory cell influx into the lung tissue and, corroborating the previous results, there were 40% lower levels of neutrophils in the pulmonary tissue of immunized animals after infection, compared to non-immunized animals (**Figure 1F**). Differences in relative quantities of macrophages and eosinophils between immunized and non-immunized groups were not observed (**Figures 1G, H**).

Indeed, EVs-immunized animals presented a decrease of approximately 30% in vascular permeability, in terms of total proteins into the BAL, compared to non-immunized animals (**Figure 1I**).

Together, these results show that the immunization with *A. fumigatus* EVs is able to modulate the inflammatory infiltrate to lung after fungus infection, characterized mainly by a decrease of neutrophils influx.

### Pre-Exposure to EVs from *A. fumigatus* Stimulates the Production of Specific IgG

To better understand the impact of *A. fumigatus* EVs on the immune response during fungal infection, we evaluated the ability of EVs to stimulate a humoral response. Therefore, we investigated the production of reactive IgG in the serum, on the 16th day after the first immunization dose. It was observed that the immunization protocol used was able to stimulate the production of reactive antibodies against *A. fumigatus* EVs (**Figure 1J**).

### EVs Immunization Reduce the Production of Inflammatory Mediators During *A. fumigatus* Infection

**Figure 2:**
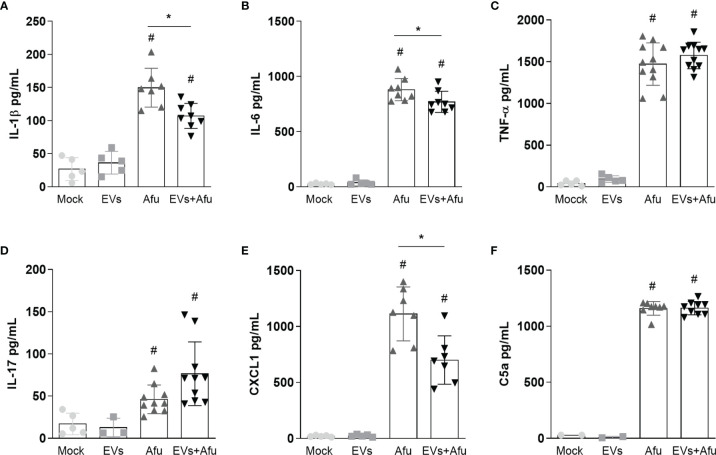
Immunization with *A. fumigatus* EVs decreases the pro-inflammatory mediator’s production during infection. C57BL/6 mice were immunized twice with 2.2 × 10^9^ EVs and challenged with 1 × 10^8^ *A. fumigatus* conidia. The inflammatory mediators IL-1β **(A)**, IL-6 **(B)**, TNF-α **(C)**, IL-17 **(D)**, CXCL1 **(E)** and C5a **(F)** presents in BAL from animals were quantified. Data are mean ± SD of N = 5-6 animals per group. #/* P < 0.05 when compared to the Mock group (#) or as indicated (*), as determined by One Way-ANOVA. Data are representative of two experiments.

Considering that EVs immunization reduced the inflammatory infiltrate cells for the lung post infection, we also investigated the levels of inflammatory mediators’ production after immunization with EVs and subsequent fungus infection. A decrease in levels of pro-inflammatory mediators, such as IL-1β and IL-6 (**Figures 2A, B**), was observed. There was no difference in TNF-α and IL-17 levels between groups (**Figures 2C, D**).

As we observed a reduction in cell infiltrate, we analyzed levels of CXCL1 and C5a, as neutrophils chemoattractant mediators. Our results demonstrate that immunization with *A. fumigatus* EVs was able to reduce CXCL1 levels in about 40% corroborating the modulation of the inflammatory response mediated by EVs (**Figure 2E**). Differences were not observed in C5a levels between immunized and non-immunized groups, post infection (**Figure 2F**).

These data suggest that the immunization with *A. fumigatus* EVs is able to reduce the inflammation into the lungs by the decrease in pro-inflammatory mediators’ production, post infection by fungus.

### Immunization With *A. fumigatus* EVs Enhances the Phagocytic Capacity and Fungal Clearance

**Figure 3:**
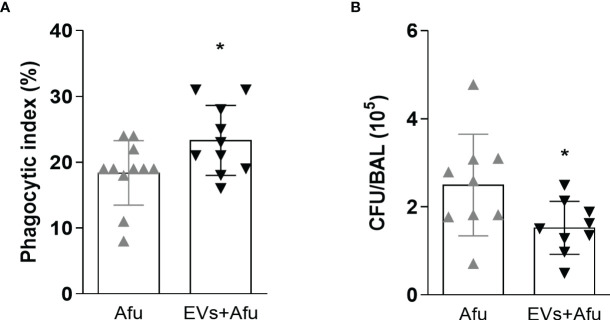
Immunization with EVs impacts in phagocytic capacity and fungal clearance. C57BL/6 mice were immunized twice with 2.2 × 10^9^ EVs and challenged with 1 × 10^8^ *A. fumigatus* conidia. Phagocytosis **(A)** and fungal killing **(B)** were evaluated in BAL. Data are mean ± SD of N = 5-6 animals per group. *P < 0.05 when compared to the Afu and EVs+Afu group, as determined by two-sample independent t-tests. Data are representative of two experiments.

As the inflammatory response was altered by immunization with EVs, we investigated if the main effectors mechanisms of immune response to fungal infection were maintained. We analyzed the phagocytic capacity and fungal clearance by infiltrate immune cells from BAL. It was observed that *A. fumigatus* EVs were able to increase phagocytic capacity of immune cells into the airways of about 20%, compared to non-immunized mice (**Figure 3A**). Moreover, the effectivity of phagocytosis, evaluated as clearance of fungal burden, was also analyzed. The increase in phagocytosis was accompanied by a reduction in fungal burden into the lungs of infected animals (**Figure 3B**). These results demonstrate that the immunization of mice with *A. fumigatus* EVs is able to impact the phagocytic capacity and consequently in fungal clearance during infection.

### Immunization With *A. fumigatus* EVs Reduces Pulmonary Tissue Damage After Fungus Infection

In order to evaluate if the inflammatory alterations observed after infection of *A. fumigatus* EVs-immunized animals influenced the outcome of infection into the lung tissue, we analyzed histopathological architecture.

**Figure 4:**
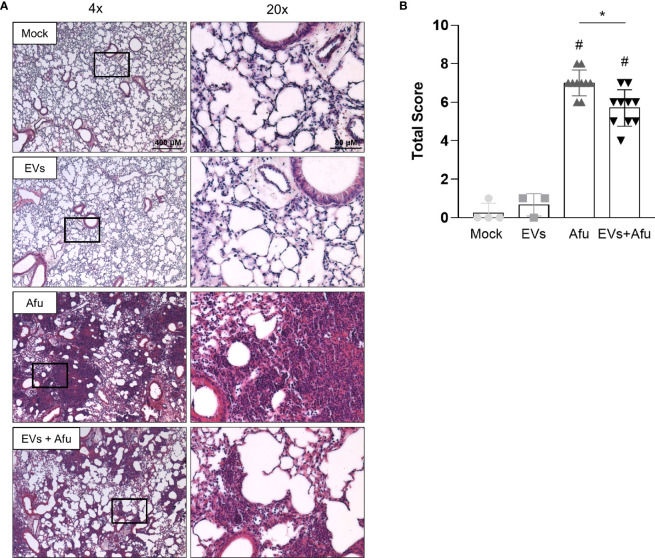
Immunization with *A fumigatus* EVs attenuates the lung damage post infection. C57BL/6 mice were immunized twice with 2.2 × 10^9^ EVs and challenged with 1 × 10^8^ *A. fumigatus* conidia. Representative slides of hematoxylin and eosin (H&E) stained lungs are shown **(A)**. Scale bars = 400 µm (low magnification) and 80 µm (high magnification). Right slides are higher magnifications (20x) of the selected areas (boxes) in left slides (4x). Histopathological score evaluated airway, vascular, and parenchymal inflammation, neutrophilic infiltration, and epithelial injury **(B)**. Data are mean ± SD of N = 5-6 animals per group. ^#^/*P < 0.05 when compared to the Mock group (^#^) or as indicated (*), as determined by One Way-ANOVA. Data are representative of two experiments.

Infection with *A. fumigatus* promoted robust changes in lung tissue. Non-immunized animals showed a strong inflammatory infiltrate, with neutrophils predominance, widely distributed in all pulmonary parenchyma. The cell infiltrate showed a multifocal and coalescent pattern, with partial loss of tissue architecture. In some regions, it was possible to observe cellular/tissue necrosis points and, in some cases, small bleeding areas. Interestingly, the animals that were previously immunized with EVs showed a moderate neutrophilic inflammatory infiltrate. Despite the existence of cellular focus, especially in peribronchiolar regions, it was not widely coalescent, so the tissue architecture was preserved. Taken together, these data reveal that *A. fumigatus* EVs are able to partially avoid lung tissue damage (**Figure 4**).

### Synergistic Effects of EVs Immunization and Amphotericin B Treatment During Pulmonary Aspergillosis Infection

**Figure 5:**
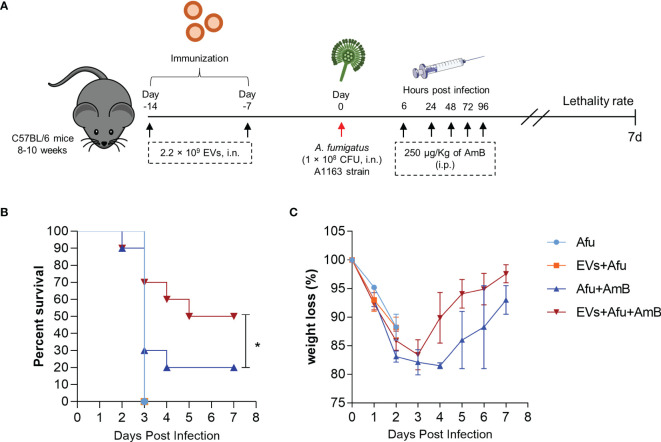
Immunization with *A. fumigatus* EVs elicits protection after treatment with AmB. C57BL/6 mice were immunized twice with 2.2 × 10^9^ EVs and challenged with 1 × 10^8^ *A. fumigatus* conidia. Mice were treated with 250 µg/Kg of AmB (i.p.) 6, 24, 48, 72 and 96 hours post infection **(A)**. The groups were monitored for lethality rates **(B)** and weight loss **(C)**. N = 10 animals per group. *P < 0.05 when compared to the Afu+AmB and EVs+Afu+AmB.

The immunization with *A. fumigatus* EVs stimulated a substantial reduction of pro-inflammatory response and recruitment of neutrophils to the site of infection. This modulatory effect in the inflammatory response did not affect the effectiveness of phagocytosis and fungal clearance into the lungs. We hypothesized that immunization with EVs could be a synergistic approach with amphotericin B (AmB) subclinical doses for pulmonary aspergillosis treatment (**Figure 5A**). Our results demonstrate that immunized animals did not show an increase in survival after infection, compared to non-immunized animals. Interestingly, animals that were immunized and treated with AmB post infection showed a 30% increase in survival, compared to AmB treated control (**Figure 5B**). This synergistic effect was accompanied with a faster recovery of animal weight loss during the course of infection, after the fourth day post infection (**Figure 5C**). These exciting results demonstrate the potential of *A. fumigatus* EVs as adjuvant strategy in AmB treatment during *A. fumigatus* infection.

## Discussion

Fungi are still underestimated as pathogens. Fungal lung infections are considered a serious clinical problem, especially in immunocompromised individuals (Rodrigues and Nosanchuk, 2020). In this sense, it is necessary to search for strategies that aim to protect these individuals from serious health complications resulting from opportunistic infections.

**Figure 6:**
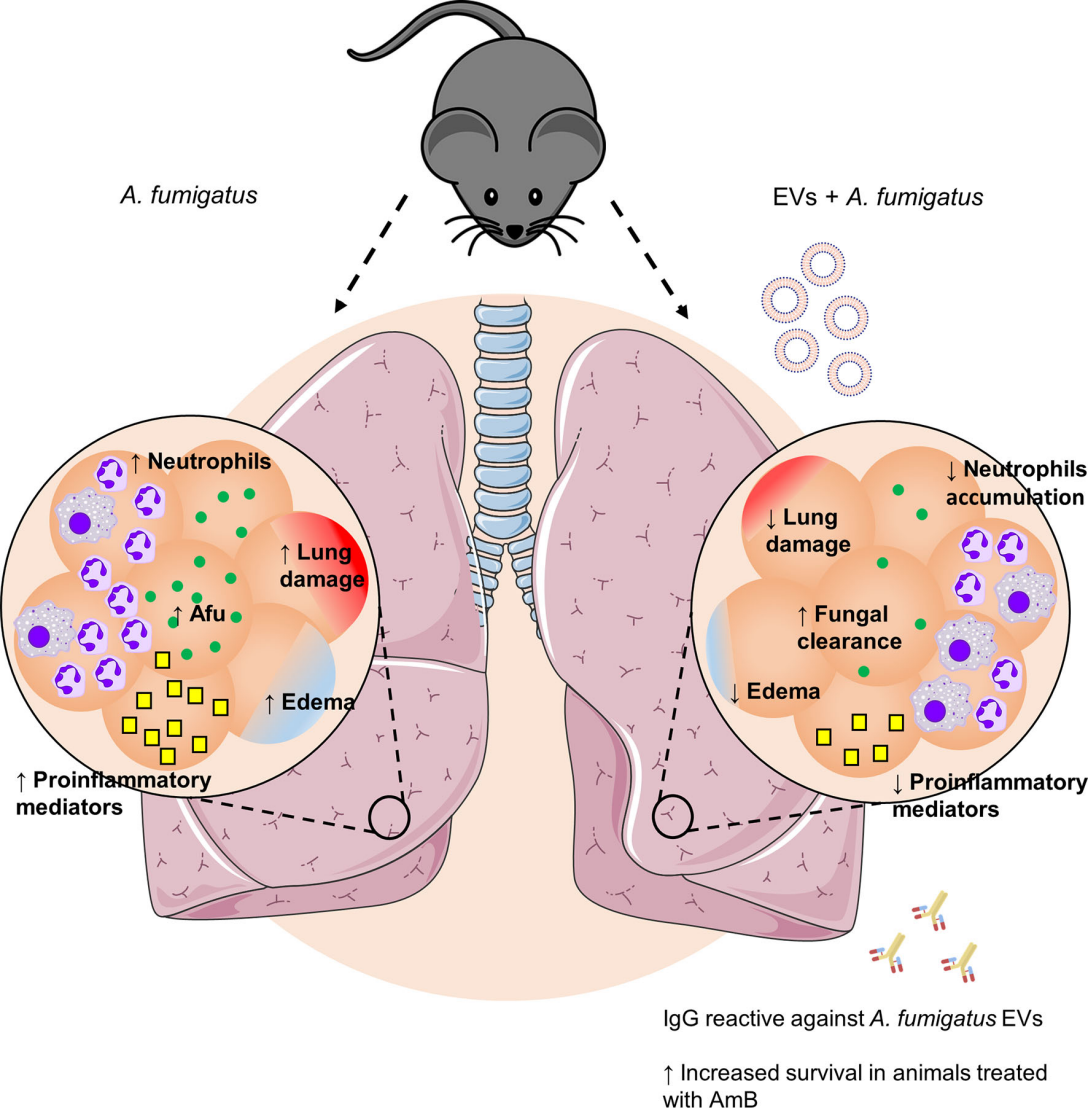
Effects of immunization with *A. fumigatus* EVs. Pulmonary infection by *A. fumigatus* promotes an increase in neutrophil infiltrate to the alveoli. The increase in the inflammatory infiltrate is supported by a growth in production of pro-inflammatory mediators, which culminates in the formation of edema and lung injury. Immunization with *A. fumigatus* EVs modulates the inflammatory infiltrate to the lungs, characterized by a reduction in the accumulation of neutrophils and in the production of pro-inflammatory mediators. In addition, it stimulates fungal clearance mechanisms. This control is mediated, in part, by the presence of specific antibodies, which, combined with treatment with low doses of AmB, are able to protect animals from a fatal infection.

EVs have represented an important tool for delivering antigens to host cells capable of stimulating the immune system in different ways (Brown et al., 2015; Rizzo et al., 2020b). Recently, it has been shown that *A. fumigatus* releases EVs containing lipids and a variety of proteins and carbohydrates (Souza et al., 2019; Rizzo et al., 2020a). The EVs of *A. fumigatus* were able to stimulate phagocytic cells *in vitro*, promoting the production of pro-inflammatory mediators and enhancing fungal clearance (Souza et al., 2019). Here we investigated the role of *A. fumigatus* EVs *in vivo*, using murine pulmonary aspergillosis. Multiple immunizations with *A. fumigatus* EVs were able to (i) decrease the neutrophilic infiltrate to the lungs; (ii) increase fungal clearance in the airways; (iii) attenuate lung damage; and (iv) reduce lethality associated with treatment with low doses of AmB after infection by the fungus (**Figure 6**).

To evaluate the effect of *A. fumigatus* EVs *in vivo*, mice were initially intranasally immunized with EV preparations; considered the main route of infection by the fungus (Dagenais and Keller, 2009). The intranasal route of administration offers the advantage of being less invasive and stimulating mucosal and systemic responses (Walkowski et al., 2021).

Infection by *A. fumigatus* can lead to an exacerbated inflammatory response, characterized by the accumulation of leukocytes and edema formation in the lungs, which can be lethal (Malacco et al., 2019a; Malacco et al., 2019b; Malacco et al., 2020). Here, we show that immunization with EVs prevented exacerbated inflammation in the lungs during infection. One of the main factors that led to this decreasing in the inflammatory infiltrate to the lungs was the diminishment in the production of the pro-inflammatory mediators IL-1β, IL-6, and CXCL1 by the immunized animals. The cytokines IL-1β and IL-6 play a crucial role in the initiation of the inflammatory response and are also responsible for inducing an increase in endothelial permeability. In addition, CXCL1 plays an important role in neutrophil recruitment. Taken together, the continuous and uncontrolled synthesis of these molecules can result in an increase in inflammatory infiltrate, tissue damage, and loss of function (Puhlmann et al., 2005; Tanaka et al., 2014; Sawant et al., 2016; Alsaffar et al., 2018; Fahey and Doyle, 2019). In this sense, our data suggest that immunization with *A. fumigatus* EVs prevents the formation of an exacerbated inflammatory response during infection, protecting the lungs from extensive damage resulting from inflammation.

Similar to our findings, mice previously immunized with *Staphylococcus aureus* EVs, and subsequently infected with a sublethal dose of the bacteria intranasally, were prevented from prominent inflammatory exudate in the lungs and systemic inflammation, compared with animals that did not receive EVs (Choi et al., 2015).

Phagocytosis is a process temporally subsequent to the activation and recruitment of cells to the site of infection. In our previous study, we saw that stimulation of phagocytic cells with *A. fumigatus* EVs, *in vitro*, was able to increase the phagocytic capacity of cells after infection with conidia (Souza et al., 2019). Here, we show that phagocytes present in the alveoli of infected mice, previously immunized with EVs, showed an increase in phagocytic capacity and fungal clearance, compared to non-immunized animals. The ability of EVs to stimulate fungal clearance mechanisms was also observed in study models with *C. neoformans*, *C. albicans*, *A. flavus*, and *P. brasiliensis* (Oliveira et al., 2010; Vargas et al., 2015; Silva et al., 2016; Vargas et al., 2020; Brauer et al., 2020; Baltazar et al., 2021).

Interestingly, we demonstrated that immunized animals produced reactive antibodies against *A. fumigatus* EVs. We have previously demonstrated that the serum of mice infected with a non-lethal dose of *A. fumigatus* is able to react against proteins present in EVs of *A. fumigatus* (Souza et al., 2019). EVs of *A. fumigatus* have several proteins involved in the physiology of the fungus and also in its virulence, such as allergens, heat shock proteins, and proteins involved in resistance to oxidative stress, suggesting that this protein charge of the EVs could act as a potential stimulator of the immune system (Souza et al., 2019; Rizzo et al., 2020a).

One of the most important molecules of *A. fumigatus* that are involved in virulence are allergens. Asp F1 and Asp F3 are among the allergens found in EVs of *A. fumigatus* (Souza et al., 2019). Asp F3 is a peroxiredoxin considered one of the most abundant proteins of *A. fumigatus*. Studies using murine models have shown that immunization with recombinant variants of Asp F3 leads to the production of specific antibodies and stimulates a protective response in immunosuppressed animals. Furthermore, immunization prevented the intense infiltration of polymorphonuclear cells in the lungs, compared to non-immunized animals (Ito et al., 2006; Ito et al., 2009). Asp F1 is a ribotoxin and considered the main allergen of *A. fumigatus*. It was shown that the Asp F1 P1 peptide was able to regulate the allergic response in animals challenged with *A. fumigatus* allergens and antigens (Chaudhary et al., 2009).

Another potential immune-stimulating protein is heat shock proteins (HSP). HSPs are involved in morphogenesis, stress response (temperature and pH), osmolarity, and antifungal resistance (Cordeiro et al., 2016; Cleare et al., 2017; O’Meara et al., 2017). This family of proteins has also been identified in other fungal EVs, such as *C. neoformans*, *Histoplasma capsulatum*, and *C. albicans* (Albuquerque et al., 2008; Rodrigues et al., 2008; Vargas et al., 2015). Studies have shown that immunization of mice with different formulations of HSP90 derived from *C. albicans* was able to induce the production of specific antibodies and protect the animals in a model of systemic candidiasis (Raška et al., 2005; Raska et al., 2008).

Additionally, *A. fumigatus* EVs have proteins involved in metabolic processes, filamentous growth, sporulation, cell cycle, and transport (Souza et al., 2019). These components are also able to stimulate the immune system and trigger protective responses. Asif and colleagues (Asif et al., 2010) showed that several *A. fumigatus* proteins were serum reactive in serum from rabbits infected with *A. fumigatus*. Among those present in EVs of *A. fumigatu*s, we can highlight phosphoglucomutase and phosphoglycerate kinase - involved in glycolysis; 1,3-beta-glucanosyltransferase gel1 - involved in cell wall remodeling; and 40S ribosomal protein S3 and 60S ribosomal protein - involved in the biosynthesis of proteins (Asif et al., 2010). Our data suggest that immunization with *A. fumigatus* EVs was able to stimulate adaptive response mechanisms that, together with effector mechanisms of innate immunity, culminated in an increase in the ability of fungal clearance and modulation of the pro-inflammatory response.

*A. fumigatus* is an opportunistic pathogen known to cause infection in immunocompromised individuals. In this study we used a previously described animal model of pulmonary aspergillosis in immunocompetent animals (Malacco et al., 2019b). Besides this model is suitable for the study of host-pathogen interactions, many other similar studies have demonstrated the stimulation of the immune cells by EVs. Vargas and colleagues (Vargas et al., 2015) demonstrated that EVs from *C. albicans* strongly induced the expression of MHC class II and CD86 costimulatory molecule on dendritic cells *in vitro* (Vargas et al., 2015). Similar results were observed with *S. aureus* EVs (Choi et al., 2015), suggesting the ability of EVs to stimulate CD4+ T lymphocytes. Recently, it has been demonstrated that animals immunized with *P. brasiliensis* EVs presented higher levels of activated CD4+ T lymphocytes in the BAL after infection (Baltazar et al., 2021). These findings reinforce that one of the immunomodulation mechanisms triggered by EVs may be the activation of a cellular response mediated by effector CD4+ T lymphocytes, which in turn amplify effective phagocytosis during infection. Interestingly, it was shown that immunization of mice with *C. albicans* EVs induced the production of specific antibodies, and in immunosuppressed animals it had a protective effect and reduced the fungal burden in the spleen, kidney, and liver (Vargas et al., 2020). Together with data in literature, our findings suggest that EVs may be effective in potentiating the immune response under immunosuppressive conditions during infection.

Finally, immunization using EVs of *A. fumigatus* combined with treatment with low doses of AmB was able to protect 50% of infected animals. AmB is one of the main antifungal drugs used, recommended mainly for the treatment of triazole-resistant strains. Despite its efficiency, the use of AmB should be cautious, since depending on its formulation and treatment frequency, this drug can confer serious nephrotoxic effects on the host. Furthermore, the emergence of drug-resistant strains can become a limiting factor for treatment (Mesa-Arango et al., 2012; Ashu et al., 2018). Lewis and colleagues (Lewis et al., 2002) demonstrated that lower doses of AmB in immunosuppressed animals are not able to protect mice in a model of invasive aspergillosis (Lewis et al., 2002). In our work, immunization with *A. fumigatus* EVs, prior to a low-dose antifungal treatment, was able to prevent exacerbated inflammatory response, contributing to the effectiveness of AmB treatment, potentiating fungal clearance. In addition, very high doses were less effective in protecting animals from *A. fumigatus* infection, leading to earlier death than animals treated with lower doses, probably due to the toxic effects of AmB (Clemons et al., 2011). Taken together, our data suggest that EVs can confer a protective response, when combined with AmB treatment, without the need of higher toxic doses of the antifungal. Additional studies should be carried out altering the vaccination strategy and the immune competence of animals in order to better understand the effects of *A. fumigatus* EVs.

In conclusion, the results generated here show that multiple immunizations with *A. fumigatus* EVs have a protective effect on inflammation and fungal proliferation in lungs of infected animals, which culminate in a reduction in lethality when using low concentrations of AmB for treatment. Moreover, EVs may serve as a complementary prophylactic alternative to protect against serious clinical complications after *A. fumigatus* infection.

## Data Availability Statement

The original contributions presented in the study are included in the article. Further inquiries can be directed to the corresponding authors.

## Ethics Statement

The animal study was reviewed and approved by Comitê de Ética no Uso de Animais - CEUA 255/2018 (Universidade Federal de Minas Gerais).

## Author Contributions

Conception of the study: FS. Designed the experiments: JS and FS. Performed the experiments: JS, IG, NM, FM and CQ-J. Interpretation of the results and data analysis: JS, IG, NM, FM, CQ-J and FS. Contributed reagents/materials/analysis tools: MT and FS. Wrote the manuscript: JS and FS. All authors read and approved the final manuscript.

## Funding

This work was supported by Pró-Reitoria de Pesquisa at the Universidade Federal de Minas Gerais, Conselho Nacional de Desenvolvimento Científico e Tecnológico - 474528-2012-0; 483184-2011-0. Fundação de Amparo à Pesquisa do Estado de Minas Gerais - APQ- 01756-10; APQ-02198-14; APQ-03950-17; APQ-01899-18. This study was financed in part by the Coordenação de Aperfeiçoamento de Pessoal de Nível Superior - Brasil (CAPES) - Finance Code 001 and Instituto Nacional de Ciência e Tecnologia em Dengue e Interação Microrganismo Hospedeiro (INCT em Dengue). The funders had no role in study design, data collection and analysis, decision to publish, or preparation of the manuscript.

## Conflict of Interest

The authors declare that the research was conducted in the absence of any commercial or financial relationships that could be construed as a potential conflict of interest.

## Publisher’s Note

All claims expressed in this article are solely those of the authors and do not necessarily represent those of their affiliated organizations, or those of the publisher, the editors and the reviewers. Any product that may be evaluated in this article, or claim that may be made by its manufacturer, is not guaranteed or endorsed by the publisher.
